# Short Photoperiod-Dependent Enrichment of *Akkermansia spec*. as the Major Change in the Intestinal Microbiome of Djungarian Hamsters (*Phodopus sungorus*)

**DOI:** 10.3390/ijms24076605

**Published:** 2023-04-01

**Authors:** Ann-Kathrin Kissmann, Frank Rosenau, Annika Herwig, Victoria Diedrich

**Affiliations:** 1 Institute for Pharmaceutical Biotechnology, Ulm University, Albert-Einstein-Allee 11, 89081 Ulm, Germany; ann-kathrin.kissmann@uni-ulm.de (A.-K.K.); frank.rosenau@uni-ulm.de (F.R.); 2 Institute of Neurobiology, Ulm University, Albert-Einstein-Allee 11, 89081 Ulm, Germany; annika.herwig@uni-ulm.de

**Keywords:** Siberian hamster, fecal sample, constant diet, seasonal acclimation

## Abstract

The Djungarian hamster (*Phodopus sungorus*) is a prominent model organism for seasonal acclimatization, showing drastic whole-body physiological adjustments to an energetically challenging environment, which are considered to also involve the gut microbiome. Fecal samples of hamsters in long photoperiod and again after twelve weeks in short photoperiod were analyzed by 16S-rRNA sequencing to evaluate seasonal changes in the respective gut microbiomes. In both photoperiods, the overall composition was stable in the major superordinate phyla of the microbiota, with distinct and delicate changes of abundance in phyla representing each <1% of all. Elusimicrobia, Tenericutes, and Verrucomicrobia were exclusively present in short photoperiod hamsters. In contrast to Elusimicrobium and Aneroplasma as representatives of Elusimicrobia and Tenericutes, *Akkermansia muciniphila* is a prominent gut microbiome inhabitant well described as important in the health context of animals and humans, including neurodegenerative diseases and obesity. Since diet was not changed, *Akkermansia* enrichment appears to be a direct consequence of short photoperiod acclimation. Future research will investigate whether the Djungarian hamster intestinal microbiome is responsible for or responsive to seasonal acclimation, focusing on probiotic supplementation.

## 1. Introduction

The last 20 years have demonstrated the great importance of the intestinal microbiome for the regulation of most physiological and many pathophysiological conditions. Intestinal microbiota affect neuroendocrine regulation and neurodegenerative disorders [1], as well as energy metabolism and obesity, one of the most threatening diseases of affluence in developed countries [2].

The change of (patho-)physiological parameters in the host can lead to profound changes in its microbiome and vice versa, whereby both the host and the microbiome respond to a multitude of environmental stimuli [3]. The seasonal cycle of the temperate zone continental climate induces remarkable but predictable environmental changes on an annual scale, influencing the life history of its inhabitants, such as the Djungarian hamsters (*Phodopus sungorus*). This species has long been studied as a model organism of seasonal acclimatization. Interestingly, the hamsters exclusively use the highly predictable photoperiod to anticipate seasonal changes of their environment, including the energetic challenges of the winter [4,5,6]. As soon as the daily light phase decreases below 13.5 h, seasonal acclimatization starts [7]. The hamsters voluntarily reduce their food intake, body and fat mass, body temperature and activity, as well as gonadal size and function to lower their overall energy expenditure. In parallel, they develop a white, highly insulating winter fur [8]. After approximately two months, the hamsters have largely reached their energy-saving winter phenotype and start to express daily torpor during their resting phase [9]. The energy-saving purpose of these changes is undeniable, and the hamsters achieve it by decreasing energy expenditure, as well as increasing energy assimilation from a given amount of food. Piscitiello and coworkers showed that the small intestine of short photoperiod-acclimated hamsters had a relatively larger mucosal surface and electrogenic glucose transport capacity compared to hamsters in a long summer-like photoperiod, presumably resulting in a body size-optimized glucose uptake capacity [10]. Regarding glucose as the major energy source, a much earlier study in mice already demonstrated that the microbiome profile also influences the intestinal energy assimilation capacity by changing glucose absorption [11].

Bailey and colleagues were the first to investigate the intestinal microbiome of Djungarian hamsters. They did not find seasonal changes in diversity and richness, but a lower relative abundance of Proteobacteria and a higher relative abundance of Firmicutes in short photoperiod-acclimated hamsters, which was attributed to annual changes in the hamsters’ natural diet [12,13]. The involvement of melatonin signaling via the pineal gland was confirmed in a later study [14]. Since 2020, researchers discovered gender-related differences in hamster microbiome changes [15], as well as a profound correlation between maternal or seasonally mediated aggression and intestinal microbiome composition [15,16].

Altogether, these findings open up several possibilities for further investigation of the Djungarian hamsters’ intestinal microbiome, which is supportive of their energy-saving winter phenotype. Since the described seasonal changes occurred despite a constant diet, the intestinal microbiome might be reorganized in anticipation of the upcoming winter season, bearing the potential to induce other acclimatization traits. This study summarizes the first analysis of the major seasonal changes in the fecal microbiome of Djungarian hamsters from the breeding colony of Ulm University in Germany. The following findings led to the development of a comprehensive project to intensify research on the characterization and manipulation of the hamsters’ seasonal microbiome, focusing on potential “acclimation-inducing probiotics.”

## 2. Results

### 2.1. Short Photoperiod Acclimation

**Figure 1:**
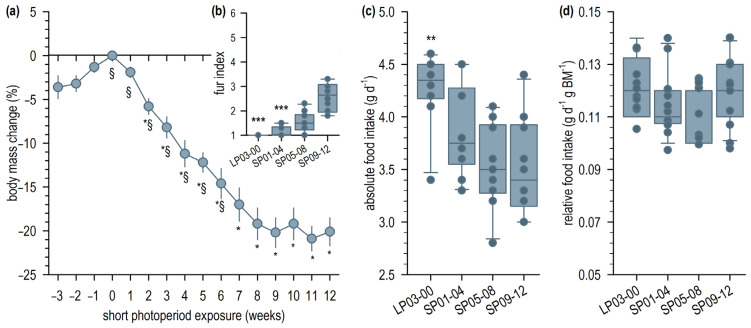
Indicators of short photoperiod (SP) acclimation. (**a**) Mean relative body mass change (±SEM) of the investigated hamster group (*n* = 10) during twelve weeks of SP exposure; One Way Repeated Measures ANOVA, *F*_(12, 108)_ = 53.6, *p* < 0.001; Bonferroni *post hoc* test with a significant difference from week 0 (*), the beginning of the SP exposure, or a significant difference from week 12 (§), shortly before sacrifice; *p* ≤ 0.05. (**b**) Median fur index (25th, 75th quartile) in four acclimation intervals, whereby LP03-00 refers to long photoperiod; Friedman ANOVA, *χ*^2^_(3)_ = 27.6, *p* < 0.001; Tukey *post hoc* test with *** marking a significant difference from LP09-12. (**c**) Median daily food intake during the four acclimation intervals; One Way Repeated Measures ANOVA, *F*_(3, 24)_ = 18.2, *p* < 0.001; Bonferroni *post hoc* test with ** marking a significant difference from all other intervals. (**d**) Median daily food intake in relation to individual body mass.

During the four weeks before the first fecal sampling, ten long photoperiod-acclimated hamsters had an average body mass of 35.7 ± 3.3 g, a summer-like fur index of 1 (Figure 1b), and a food intake of 4.3 ± 0.4 g per day (Figure 1c), corresponding to a relative food intake of 0.12 ± 0.01 g per g body mass (Figure 1d). Short photoperiod exposure for twelve weeks resulted in a decrease in body mass by 20.1 ± 5.1% down to 29.1 ± 3.2 g (Figure 1a), a decrease in absolute daily food intake to 3.5 ± 0.5 g per day (Figure 1c, SP09-12), as well as an increase in fur index to 2.5 ± 0.5 (Figure 1b). The body mass-related food intake did not change significantly (Figure 1d). The decrease in body mass was already significant after the second week of SP exposure and did not continue since week seven, i.e., the second SP interval (Figure 1a). The daily food intake dropped significantly between the LP and the first SP interval and remained stable afterwards (Figure 1c). In contrast, a significant increase of fur index was only detectable in the last SP interval between week nine and twelve (Figure 1b).

### 2.2. Short Photoperiod-Induced Change in Intestinal Microbiome

**Figure 2:**
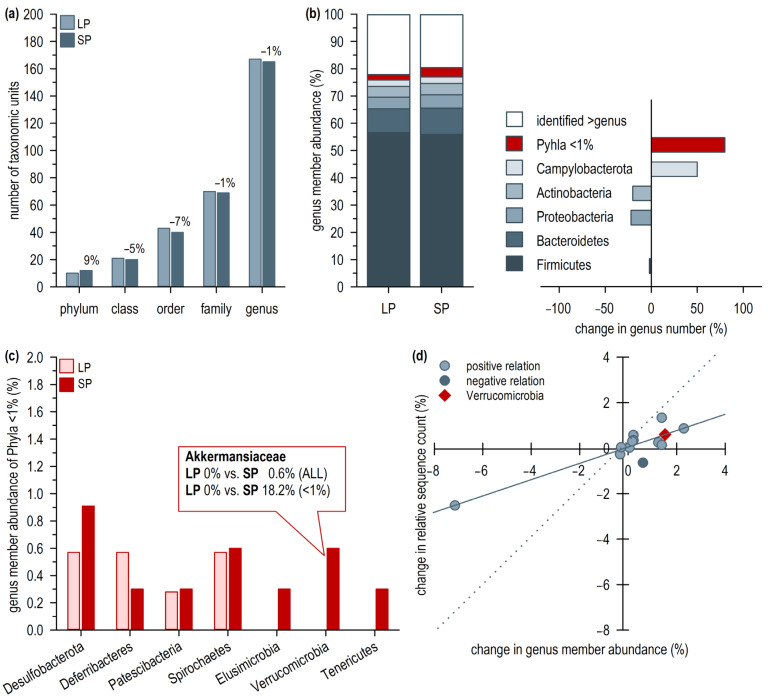
Microbiome composition during long photoperiod (LP) and short photoperiod (SP) exposure. Fecal samples of the same hamsters (*n* = 10) were taken during LP and after twelve weeks of SP exposure, whereby the individual samples were pooled to one LP and one SP sample. (**a**) Absolute number of different taxonomic units which could be identified to genus level in the LP and SP sample. Numbers above the columns represent the relative change in taxonomic unit number after twelve weeks of SP acclimation. (**b**) Relative abundance of genus members per phylum in LP and SP. Phyla with a relative abundance of ≥1% in LP are depicted separately, phyla with a relative abundance of <1% are summarized. (**c**) Relative abundance of genus members in the phyla <1% comparing the LP and the SP sample. The abundance of the Verrucomicrobia increased significantly in the SP sample (Chi^2^ test, *χ*^2^_(1)_ = 17.6, *p* < 0.001). (**d**) SP-induced change in relative sequence count per phylum related to change in genus member abundance. A positive relation, i.e., phyla in the first and third quadrant, indicates that the relative sequence count increased or decreased together with the genus member abundance. A negative relation, i.e., phyla within the second and fourth quadrant (dark circle), shows that sequence count and genus member abundance change in opposite direction. The diamond marks the Verrucomicrobia including the Akkermansiacaeae. The lines mark a significant correlation between change in relative sequence count and genus member abundance, including all phyla (solid line; Spearman Rank Correlation, *r*_(13)_ = 0.7, *p* = 0.014) and only positively related phyla (dashed line; Spearman Rank Correlation, *r*_(13)_ = 0.8, *p* < 0.001).

The analysis of the intestinal microbiome of the ten representative long photoperiod-acclimated Djungarian hamsters revealed 352 taxonomical units from 18 identified phyla and 1 unidentified phylum (Figure S1e–h). As only 32% of these units could be identified down to species level, the assignment to a specific higher taxonomic level happened only for units identified at the genus level. Thus, the phyla Cyanobacteria (2 units), Armatimonadetes (1 unit), Chloroflexi (1 unit), Margulisbacteria (1 unit), Nitrospirae (1 unit), Sva0485 (1 unit), and TX1A-33 (1 unit) were excluded. Accordingly, the data set comprised units of 167 genera, 70 families, 43 orders, 21 classes, and 10 phyla (Figure 2a). Apart from 22.2% of units that were not identified on the genus level, 56.6% belonged to the Firmicutes, 8.8% to the Bacteroidetes, and less than 5% to the Proteobacteria, the Actinobacteria, the Campylobacterota, or other phyla (Figure 2b and Figure S1a–d).

In response to twelve weeks of short photoperiod exposure, the relative abundance of genus members did not change significantly among the phyla accounting for more than 1%, although the abundance of units not identified at genus level slightly decreased to 19.6% (Figure 2b). The number of genus members per class, order, and family was slightly reduced; however, the number of genus members per phylum was increased by 9%, as three new phyla emerged above detection level in the SP fecal sample, namely the Elusimicrobia, the Tenericutes, and the Verrucomicrobia (Figure 2a,c).

Especially the emergence of the Verrucomicrobia occurred due to two genus members, both belonging to the Akkermansiaceae (Figure 2c). With 0.6% of all genus members, the increase was not significant; however, related to all phyla with a <1% abundance, the increase from 0% to 18.2% was significant (Figure 2c). For the Verrucomicrobia and almost all other identified phyla, the change in genus member abundance per phylum was positively related to the change in relative sequence count, i.e., an increase in genus members was associated with an increase in relative sequence count (Figure 2d). Alternatively, a decrease in unidentified genus members was related to a decrease in their relative sequence count.

The Firmicutes were an exception, as their genus member abundance increased by 0.6% while their respective relative sequence count decreased by 0.6% (Figure 2d). The Shannon Diversity Index revealed an almost identical genus member diversity comparing the short and the long photoperiod sample (H_SP_ = 4.59 vs. H_LP_ = 4.68), although a short photoperiod-induced 50% (2 to 3) and 80% (5 to 9) increase in genus number within the Campylobacterota and <1% Phyla were detected, respectively. The larger phyla of the Firmicutes and Bacteroidetes remained rather stable (Figure 2b).

## 3. Discussion

The first characterization of the intestinal microbiome in the Djungarian hamsters of Ulm University (Germany) was performed with pooled fecal samples of ten adult hamsters acclimated to summer-like long photoperiod. To further identify respective seasonal changes, samples were taken again from the same hamsters after twelve weeks of acclimation to a winter-like short photoperiod. Despite a constant diet, the microbiome changed in response to the short photoperiod, whereby differences were not detected in the overall diversity or richness, but in the higher number of phyla and an emergence of distinct taxa, e.g., *Akkermansia muciniphila*. This begs the question whether this species might be considered a marker bacterium or even a “acclimation-inducing probiotic” in the future.

The hamsters showed the expected short-photoperiod acclimation degree and variability of changes in body mass, food consumption, and fur index [17]. Thus, these animals provided representative fecal samples to characterize and compare a long and short photoperiod intestinal microbiome in the same individuals. In line with previous studies [18], Djungarian hamsters of the Ulm University colony showed a diverse intestinal microbiome. Ignoring the taxonomic level of identification, a total of 20 phyla could be differentiated. Using the premise of identification to genus level, nine phyla remained to characterize the long photoperiod microbiome. Although this analytic strategy excluded taxonomic units identified to only phylum, class, or order level, the diversity estimated via the Shannon Index was comparable to earlier data from this species [15,18]. As in other studies from rodents and primates [19,20,21,22,23], Firmicutes and Bacteroidetes were the most abundant phyla, although the relative amount of Bacteroidetes was lower in the hamsters of the present study [12,14,18,19]. Furthermore, the Ulm hamsters’ microbiome contained a similar amount of Proteobacteria when compared to other Djungarian hamsters and other rodents, ranging from 2 to 10% [12,14,18,20,21,24,25].

Both Firmicutes and Bacteroidetes are involved in the intestinal fermentation process and an increase in their ratio could be associated with obesity in mice and humans [23,26]. Fecal transplantation from obese, leptin-deficient mice induced obesity in lean mice [27], emphasizing the great influence of the intestinal microbiome composition on energy metabolism. In the present study, the Firmicutes-to-Bacteroidetes ratio of 6.4 in long photoperiod-acclimated hamsters was comparable to obese mice [26], suggesting an “obese microbiome” in the hamsters’ high fat and leptin-resistant summer phenotype [28]. However, it contradicts earlier studies that reported lower ratios between 1.5 and 3.0 due to slightly lower Firmicutes and higher Bacteroidetes ratios [12,14,18].

In the seasonal context, the data of the present study show another similarity to earlier mouse data [26], since exposure to a short photoperiod appeared to slightly decrease the ratio to 5.8 driven by a decrease in Firmicutes and an increase in Bacteroidetes. Thus, the heavy long photoperiod-acclimated hamsters are likely comparable to obese mice, while the light and lean short photoperiod-acclimated hamsters resemble the intestinal microbiome phenotype of lean mice. In addition, a study in C57BL/6 mice demonstrated a lower ratio in animals housed in short photoperiod compared to long photoperiod, associated with a lower food intake but not body mass [25]. Earlier studies in Djungarian hamsters do not support this finding but show a short photoperiod-induced enrichment in Firmicutes [14,15] or a photoperiod-independent negative correlation between Firmicutes abundance and body mass, possibly contributing to an increased energy assimilation capacity of the winter phenotype [12,27]. The ambiguity of the present data could result from the sample pooling, but also from different housing and feeding conditions, as well as sample type (fecal or cecal) and duration of the short photoperiod exposure [12,14,15,29].

In the present study, the Proteobacteria did not change in response to short photoperiod. In a previous study, a short photoperiod-induced decrease in Proteobacteria was found, which was positively correlated with the hamsters’ body mass [12]. This result was attributed to an anticipatory response to the decreasing availability of high fat seeds in the hamsters’ natural diet during winter, occurring despite a constant diet in captivity. This idea is supported by a study showing an increase of Proteobacteria in response to a high fat diet in mice [30]. Furthermore, a very recent study in Djungarian hamsters revealed enrichment of the Proteobacteria genus Desulfovibrio in short photoperiod-acclimated Djungarian hamsters having received a fecal transplant from a long photoperiod-acclimated donor [31]. The hamsters of the present study were supplemented with sunflower seeds once per week, which might explain the stable abundance of Proteobacteria during short photoperiod acclimation. These key findings of seasonal change in dominant phyla of the microbiome emphasize that it remains to be examined whether Djungarian hamsters possess a pathological obese microbiome in long photoperiod or whether their naturally occurring seasonal changes in body and fat mass are suitable to reconsider them as models for human obesity research [32].

All other hamster studies have in common that seasonal acclimation of the intestinal microbiome occurred predominantly in distinct taxa without changing the overall microbial diversity and richness. Most of these taxa belonged to the Firmicutes, Bacteroidetes, or Proteobacteria [12,14,15,31]. Two studies also reported changes in the less abundant Patescibacteria and Tenericutes [15,31]. In accordance, the present study revealed an increase in Desulfobacterota, a decrease in Deferribacteres, as well as an emergence of Elusimicrobia and Tenericutes. Among these phyla with a relative abundance of <1%, the strongest and most significant increase was detected in the Verrucomicrobia, resulting from the enrichment of an unspecified unit of the genus Akkermansia, but also of *Akkermansia muciniphila*.

This Gram-negative, strictly anaerobic species lives on the degradation of mucin as an endogenous product of the mammalian intestine [33] and has been investigated for its positive influence on metabolic disorders, such as obesity as well as diabetes mellitus [34,35]. Consequently, *A. muciniphila* has become one of the most prominent health-related intestinal symbionts and has been used as pre- or probiotic [36]. In overweight and obese humans, *A. muciniphila* supplementation resulted in reduced levels of relevant blood markers for inflammation and liver dysfunction [37,38,39]. In mice, even pasteurized *A. muciniphila* alleviated diet-induced obesity via a complex systemic change in energy metabolism. Animals supplemented with *A. muciniphila* showed a reduced body and fat mass gain on a high fat diet without changing food intake, possibly driven by enhanced energy expenditure and fecal energy excretion combined with decreased food energy efficiency [40]. The results of the present study fit into the picture, assuming that hamsters in the long photoperiod resemble an obese phenotype with a low abundance of *A. muciniphila*. This phenotype becomes lean and energy-efficient during short photoperiod-induced winter acclimation, potentially by *A. muciniphila* enrichment.

The change in diet quality and quantity is one of the most effective parameters to change the intestinal microbiome, although many bacterial species, such as *A. muciniphila,* live on substrates produced by the host [41]. In this context, hibernators serve as valuable model organisms, as they show pronounced changes in their feeding behavior on a long-term seasonal scale, i.e., feeding and fattening during the late summer as well as fall and fasting during their prolonged hibernation bouts, with a drastic decrease in metabolism and body temperature [42,43]. During the interbout arousals, some species use energy from external food stores, while others rely on their internal fat stores only [44]. The profound morphological and physiological changes during seasonal acclimation and heterothermia severely influence the intestinal microbiome. In fat-storing thirteen-lined ground squirrels (*Ictidomys tridecemlineatus*), hibernation-associated fasting increased the abundance of mucin-degrading Verrucomicrobia as well as Bacteroidetes, and decreased the cecal abundance of Firmicutes, preferring dietary polysaccharides [45]. Similar results were obtained in the fat-storing arctic ground squirrel (*Urocitellus parryii*), where Akkermansia enrichment in particular resulted in an overall increase in Verrucomicrobia abundance [21]. Interestingly, in food-storing Syrian hamsters (*Mesocricetus auratus*), cecal abundance of *A. muciniphila* was only increased in fasted active hamsters but not in hibernating hamsters [24]. These results suggest that the enrichment arises from a combination of seasonal acclimation and dietary changes, which are difficult to separate. However, although Djungarian hamsters neither fasted nor overfed during short photoperiod acclimation, their microbiome showed an increase in Akkermansia abundance. Together with the fact that no Akkermansia blooming was detectable in seasonally hibernating carnivorous Brown bears (*Ursus arctos*) [46] or frugivorous furry-eared dwarf lemurs (*Cheirogaleus crossleyi*) [22], the results of the present study suggest a large variety of microbiome responses to a seasonally changing environment.

Summing up, the present pilot study on the characterization of seasonality in the intestinal microbiome of Djungarian hamsters from Ulm University provides evidence for the natural existence of a seasonally rather than dietary-induced obese and lean microbiome in one and the same species, together with a diet-independent enrichment of *Akkermansia muciniphila* during winter-like short photoperiod acclimation. These preliminary data set the scientific basis for future in-depth research on *A. muciniphila* as a potential “acclimation-inducing probiotic,” including (a) energy metabolism, voluntary or forced body mass changes and daily torpor expression, as well as (b) neuroendocrine regulation underlying the bidirectional communication between the host and the microbiome via the brain–gut axis [47].

## 4. Materials and Methods

### 4.1. Animals and Housing

The study investigated ten adult male Djungarian hamsters (*Phodopus sungorus*), born and raised at the Institute of Neurobiology at Ulm University in Germany according to the animal welfare law (z.231-1, Regierungspräsidium Tübingen, Germany). The hamsters were housed in an artificial long photoperiod with 16 h of light and 8 h of darkness, as well as an ambient temperature of 19 ± 2 °C and a relative humidity of 50 ± 15%. From six weeks of age, the animals were kept individually in Type III EU standard cages (area: 820 cm^2^), equipped with wood shavings and cotton tissue as nesting material. The hamsters received food (Altromin hamster breeding diet 7014, Lage, Germany) as well as tap water ad libitum and were supplemented with sunflower seeds, oat flakes, and cucumber once per week.

### 4.2. Short Photoperiod Acclimation and Sampling

At the beginning of the experiment, the hamsters had an average age of 5 ± 1 months. Their body mass and food intake as well as fur index were measured weekly over four weeks in long photoperiod.

In order to induce short photoperiod acclimation, the hamsters were then transferred to a light regime with 8 h of light and 16 h of darkness. Just before the change, a fecal sample was collected from each hamster during the weighing procedure and stored at −80 °C. From then on, the weekly measurement of body mass, food intake, and fur index continued for another twelve weeks, whereby a fur index of 1 accounted for a grey-brown summer fur, and a fur index of 6 for a white, highly insulating winter fur [48].

With the beginning of short photoperiod week 13, the hamsters were sacrificed via CO_2_ inhalation four hours after the beginning of the light phase. Directly after sacrifice, another fecal sample was collected per hamster and stored at −80 °C. In addition, diverse organ samples were collected and stored at 80 °C until further use. All experimental procedures were approved by the regional council according to the animal welfare law (Regierungspräsidium Tübingen, Germany; o.231-3).

### 4.3. 16S-rRNA Next Generation Sequencing

Fecal samples were pooled to one long photoperiod as well as one short photoperiod sample and sent to BIOMES laboratory (Wildau, Germany) to be analyzed for bacterial abundance via 16S-rRNA Next Generation Sequencing. The sequencing was performed using INTEST.pro (Biomes Laboratory, Wildau, Germany), according to Lilja and coworkers [49]. In brief, the microbial genomic DNA was extracted by bead-beating technique and the V3–V4 region of the 16S rRNA gene [50] was amplified and subsequently sequenced on the Illumina MiSeq platform using a 2 × 300 bp paired-end protocol (Illumina, San Diego, CA, USA). Normalized counts (abundance) were calculated from the raw count by biological normalization of the copy number. The relative abundance was then normalized to 100%, the resulting data space was transferred to a 0 to 1 value space [49], and the absolute as well as relative sequence count for each taxonomical unit was provided.

## Data Availability

The data presented in this study are openly available on FigShare at https://doi.org/10.6084/m9.figshare.22435057.

## Supplementary Materials

The following supporting information can be downloaded at: https://www.mdpi.com/article/10.3390/ijms24076605/s1.
