# Supplementary material for: Short Photoperiod-Dependent Enrichment of Akkermansia spec. as the Major Change in the Intestinal Microbiome of Djungarian Hamsters (Phodopus sungorus)
Source: Int J Mol Sci. 2023 Apr 1;24(7):6605. doi: 10.3390/ijms24076605 (PMC10095574; doi:10.3390/ijms24076605)
Supplement: Supplementary file 1 [file ijms-24-06605-s001.zip › ijms-2250945-supplementary.pdf]

International Journal of
Molecular Sciences

MDPI

Communication

Short Photoperiod-Dependent Enrichment of Akkermansia *spec.* as the Major Change in the Djungarian hamster (*Phodopus sungorus*) Intestinal Microbiome

Ann-Kathrin Kissmann, Frank Rosenau, Annika Herwig and Victoria Diedrich

Supplement S1

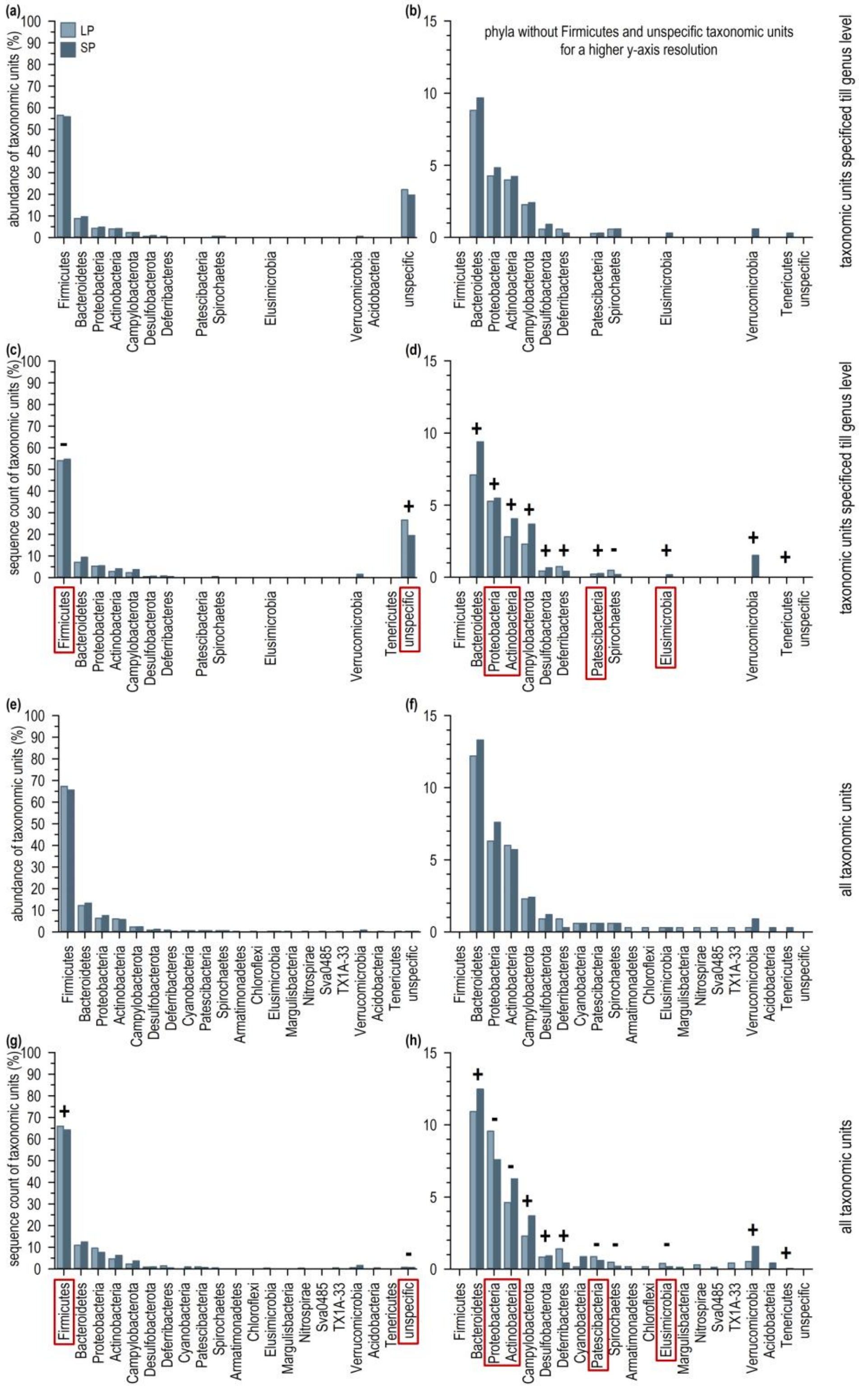

Figure S1. Abundance of taxonomic units and respective relative sequence count in two pooled fecal samples of ten Djungarian hamsters (*Phodopus sungorus*) in long photoperiod (LP, 16 h light, 8 h darkness) and again after twelve weeks of exposure to short photoperiod (SP, 8 h light, 16 h darkness) to induce winter acclimation. **(a)** and **(b)** show the abundance of all taxonomic units identified till genus level. The respective relative sequence count is shown in **(c)** and **(d)**. **(e)** and **(f)** as well as **(g)** and **(h)** summarize all sequenced taxonomic units of both sample pools and their relative sequence counts, regardless of the taxonomic identification level, resulting in a higher number of different phyla. However, the single units belonging to the Margulisbacteria, the Sva0485, or the TX1A-33 of the LP sample were only identified on the phylum level, the Armatimonadetes and the Chloroflexi on class level. In the SP sample, the unit belonging to the Acidobacteria was identified on class level only. In both samples, the Cyanobacteria units were identified on the family level, as were the Verrucomicrobia in the LP sample. In **(b), (d), (f), and (h)**, the Firmicutes and the unspecific until have been removed to improve the visualization of the phyla with a lower abundance. The + and – over the respective sequence count columns shall mark whether the direction of SP-induced change in relative sequence count was positively (+) or negatively (-) related to the direction of change in the taxonomic until abundance. The red rectangle highlights those phyla, were the relation between the two parameters was different when analyzing either all units (**(e) – (h)**) or only those units, identified to genus level (**(a) – (d)**).
